# An Improved CRISPR/Cas9 System for Genome Editing in *Populus* by Using Mannopine Synthase (MAS) Promoter

**DOI:** 10.3389/fpls.2021.703546

**Published:** 2021-07-12

**Authors:** Yi An, Ya Geng, Junguang Yao, Chun Wang, Juan Du

**Affiliations:** ^1^State Key Laboratory of Subtropical Silviculture, School of Forestry and Biotechnology, Zhejiang A&F University, Hangzhou, China; ^2^State Key Laboratory of Rice Biology, China National Rice Research Institute, Chinese Academy of Agricultural Sciences, Hangzhou, China; ^3^College of Life Sciences, Zhejiang University, Hangzhou, China

**Keywords:** poplar 84K, CRISPR/Cas9, 35S promoter, MAS promoter, *PagPDS* gene, high efficiency

## Abstract

Gene editing technology in woody plants has great potential for understanding gene function, and altering traits affecting economically and ecologically important traits. Gene editing applications in woody species require a high genome editing efficiency due to the difficulty during transformation and complexities resulting from gene redundancy. In this study, we used poplar 84K (*Populus alba* × *P. glandulosa*), which is a model hybrid for studying wood formation and growth. We developed a new CRISPR/Cas9 system to edit multiple genes simultaneously. Using this system, we successfully knocked out multiple targets of the PHYTOENE DESATURASE 8 in poplar. We found the mutation rate of our CRISPR/Cas9 system is higher (67.5%) than existing reports in woody trees. We further improved the mutation rate up to 75% at editing sites through the usage of the mannopine synthase (MAS) promoter to drive Cas9. The MAS-CRISPR/Cas9 is an improved genome-editing tool for woody plants with a higher efficiency and a higher mutation rate than currently available technologies.

## Introduction

Poplars (*Populus*) have tremendous economic and ecological value because of their timber, bioenergy applications, rapid pulp production rotation, as well as their key ecological roles in temperate forests across the northern hemisphere (Jansson and Douglas, 2007; Polle et al., 2013). With a modest genome size, high levels of genetic diversity, and a rapid growth rate (Wullschleger et al., 2002), poplars became a model system for woody plants. Hybrid poplars have been genetically modified for studying wood formation, including perennial secondary vascular cambium activity and secondary cell wall deposition (Qiu et al., 2019). Despite existence of some promising approaches for creating knockout mutants in *Populus* (Fan et al., 2015; Zhou et al., 2015; An et al., 2020a,b; Wang et al., 2020), large-scale gene mutational resources are still lacking for poplars, motivating us to build a robust and high-efficient gene editing system for *Populus*.

Currently, CRISPR/Cas9 technology is the most effective gene editing technology. In plants, the CRISPR/Cas9 system has not only been used in the study of gene function, but also plays an important role in the improvement of plant traits. Woody plants with long-life spans and outcrossing mating systems are difficult subjects for traditional mutagenesis methods (Bewg et al., 2018). Currently, there are about ten woody plants species that have been successful edited using CRISPR/Cas9 technology: apple (Nishitani et al., 2016), citrus (Jia et al., 2017), grape (Nakajima et al., 2017), cassava (Odipio et al., 2017), cacao (Fister et al., 2018), coffee (Breitler et al., 2018), kiwifruit (Wang et al., 2018), parasponia andersonii (Van Zeijl et al., 2018), pomegranate (Chang et al., 2019), and poplar (Fan et al., 2015; Zhou et al., 2015; Wang et al., 2020). Three kinds of *Populus* have been edited via CRISPR/Cas9, including *Populus tomentosa Carr* (Fan et al., 2015), 717 (*Populus tremula* × *P. alba*) (Zhou et al., 2015), and Shanxin yang (*Populus davidiana* × *P. bolleana*) (Wang et al., 2020). Extensively cultivated in China and Korea, the hybrid clone 84K (*Populus alba* × *P. glandulosa*) is a model hybrid for the study wood formation and stress response and also has relatively high rates of transformation compared with other woody plants (Li et al., 2017; Qiu et al., 2019). The recent availability of poplar 84K genome sequence facilitates functional genomic studies and the ability to design CRISPR/CAS9 approaches to target specific genes or even multiple genes (Huang et al., 2020). Therefore, it is of high priority to develop a high-efficiency gene editing system in the 84K poplar genotype.

To develop a simple and efficient gene-editing system for poplar 84K, and for editing multiple genes, we used the existing conventional isocaudomer technique using constructs based on pC1300-Cas9 (binary vector) and SK-gRNA (intermediate vector driven by AtU6-26). The system has the potential to edit an unlimited number of target genes simultaneously (Wang et al., 2015). The promoter is the key to driving the expression of transformed genes. Previous studies conducted on tobacco and maize indicated that the *mannopine synthase* (*MAS*) promoter could increase the direct expression of β-glucuronidase activity to 2–20 fold compared with the commonly used enhanced CaMV 35S promoter (Ni et al., 1995; Lee et al., 2007). We hypothesized that MAS promoter could similarly drive Cas9 to enhance editing events in 84K. Therefore, we constructed a new gene editing system which used 35S/MAS promoter to drive Cas9 and studied the editing efficiency in poplar 84K. The new system successfully editing multiple targets of phytoene desaturase gene 8 (*PDS*) with high mutation rates.

## Materials and Methods

### Cloning of *PagPDS* Fragments and Selection of Target Site

**Figure 1:**
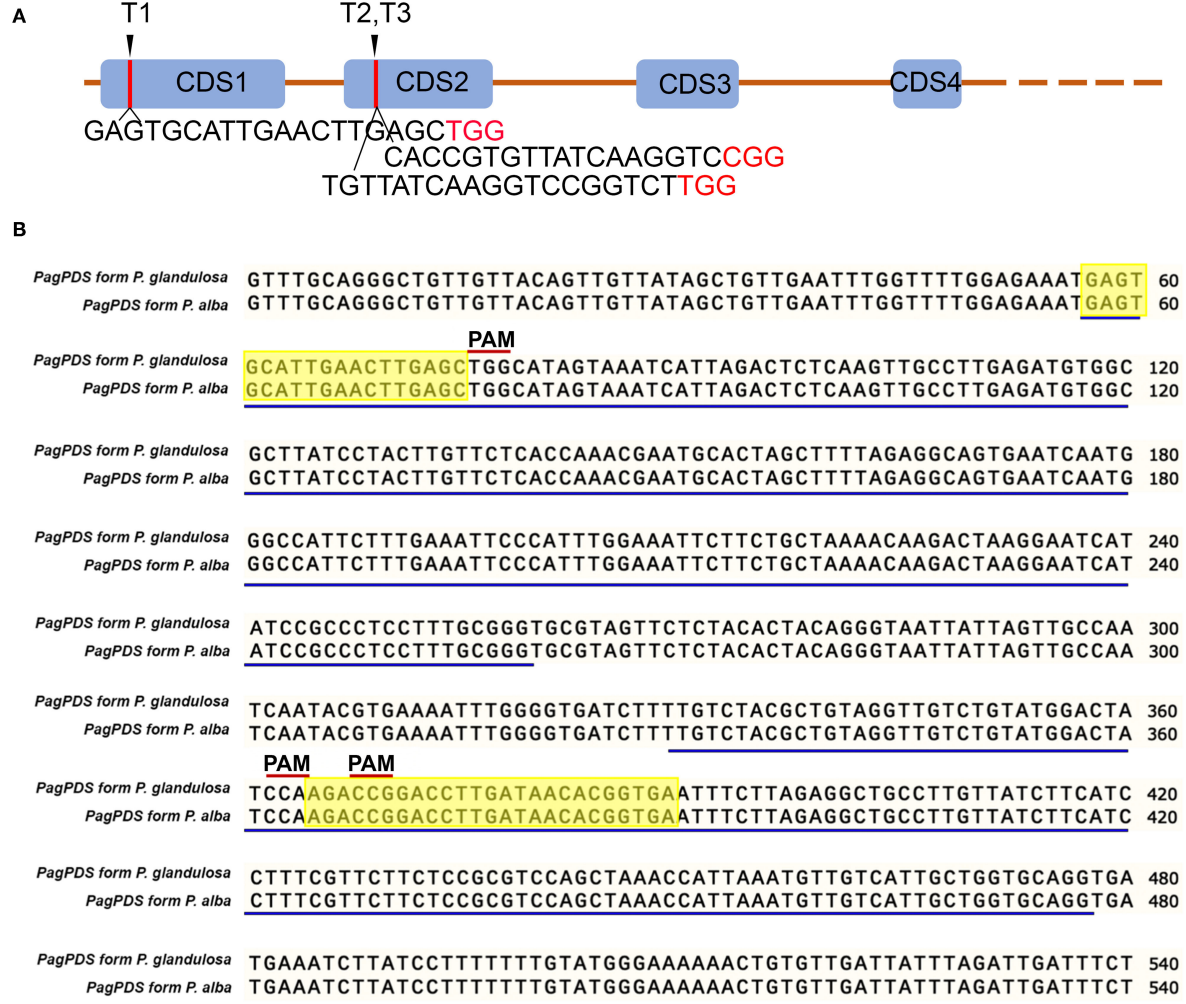
Schematic diagrams illustrating the *PagPDS* target sites and *PagPDS* gene sequence alignment. **(A)** Schematic diagrams illustrating the *PagPDS* target sites (T1–T3) and their sequences. The PAM (5′-NGG-3′) are denoted in red. Blue boxes indicate exons (exon1–exon4). Orange lines indicate introns. **(B)** *PagPDS* genomic sequences (exon1–exon2) from 84K (*Populus alba* × *P. glandulosa*). Blue lines indicate exons, letters in red indicate PAM sequence, highlighted indicate the 3 target sites.

Wide-type (WT) poplar 84K genomic DNA was extracted by the cetyltrimethylammonium bromide (CTAB) method (Fan et al., 2015). The phytoene desaturase gene (*PDS*) was cloned from extracted genomic DNA. Briefly, primers were designed against *PagPDS* from genome sequence of poplar 84K. The primers are PagPDS_F: GTTTGCAGGGCTGTTGTTACAGTT and PagPDS_R: CATTTAATGGTGCAGGGAGAACTTCAG. The amplification reaction was conducted at 95°C for 5 min, 35 cycles at 95°C for 30 s, 58°C for 30 s, 72°C for 35 s, and 72°C for 10 min. The amplicon product was electrophoresed on an ethidium bromide-stained agarose gel (1%). DNA was extracted from gel using the TIANgel Midi Purification Kit (Tiangen, Beijing, China) and cloned into the pMD18-T Simple vector and then confirmed by Sanger sequencing. The single-guide RNA (sgRNA) sequence for *PDS* was designed (Figure 1A) based on the allelic variation of *PagPDS* in Figure 1B. Three sgRNAs were designed to target three conserved sites optimizing GC content and PAM sequence.

### Vector Construction

The system is comprised of two vectors, the first of which is pC1300-Cas9, which was based on the pCAMBIA1300 backbone, which has been frequently used for *Agrobacterium-mediated* transformation in *Populus*. The vector contains the codon-optimized *Streptococcus pyogenes* Cas9 gene driven by the 2xCaMV35S promoter. pC1300-Cas9 contains *Kpn* I and *BamH* I restriction enzyme sites, which are designed for allowing integration of gRNA cassettes. Then, we developed novel vectors based on pC1300-Cas9 backbone, introducing the Superpromoter to drive Cas9 expression, with the aim to increase the expression level of Cas9 in the *Populus* cells. The Superpromoter is a synthetic promoter consists of a trimer of the octopine synthase transcriptional activating element affixed to the mannopine synthase2# transcriptional activating element plus minimal promoter (Lee et al., 2007). The MAS promoter sequence is shown in Supplementary Sequence 1.

**Figure 2:**
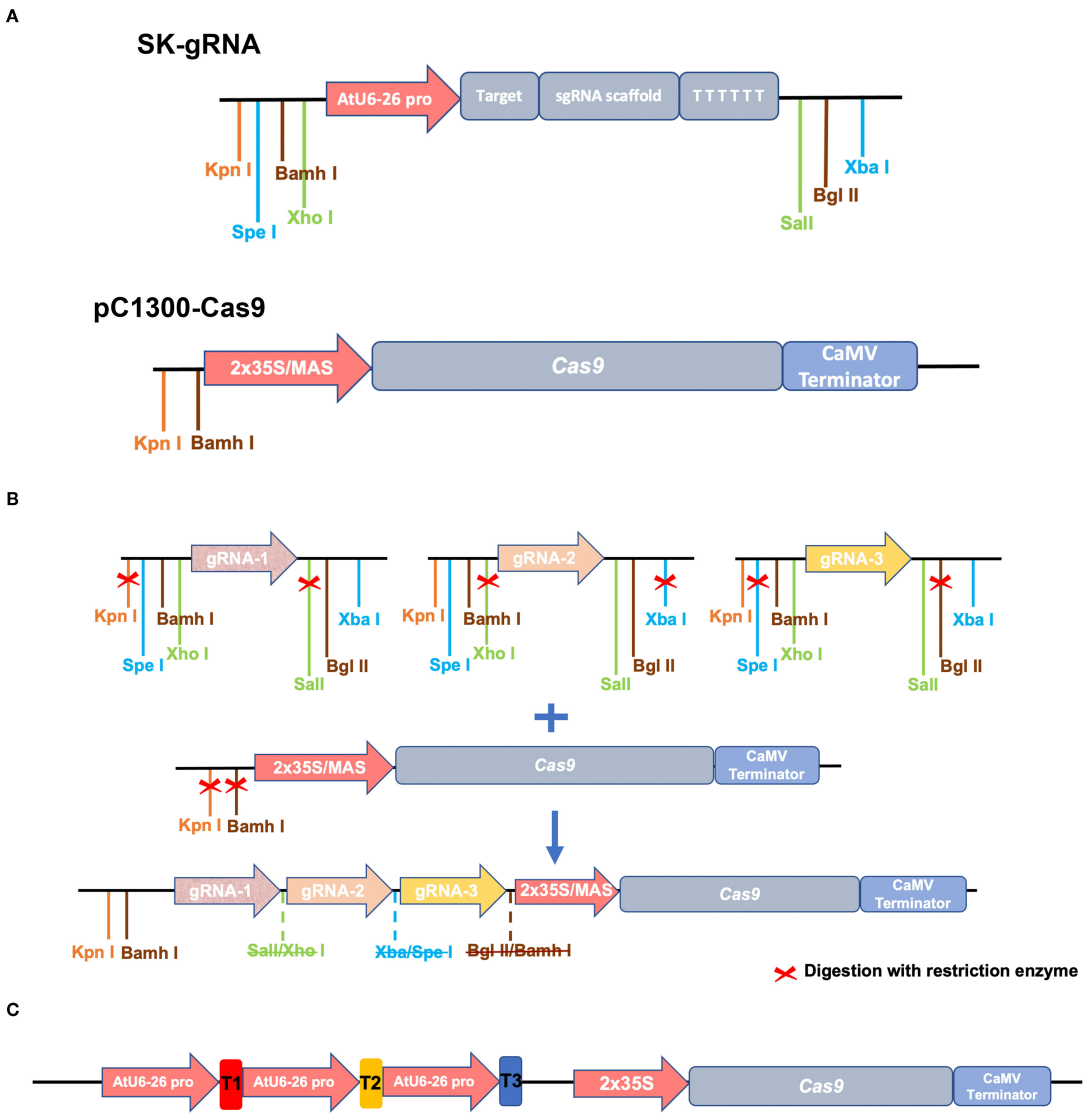
Schematic diagram illustrating the structure of vectors and construction strategy. **(A)** The intermediate vector SK-gRNA contains the U6 promotor, an sgRNA scaffold, and the terminator. The restriction sites used for cloning are indicated. Binary vector pC1300-Cas9 contains the 2 × 35S or MAS promotor, a codon-optimized Cas9 coding sequence, and the CaMV terminator. **(B)** Vector construction strategy. *Kpn* I and *BamH* I sites are indicated in front of the 2 × 35S or MAS promotor. Three gRNA scaffolds with *PagPDS* targets are, respectively, digested (contains *Kpn* I, *BamH* I, *Xho* I, *Sal* I, *Xba* I, and *Spel* I) and cloned into pC1300-Cas9 between the *Kpn* I and *BamH* I sites in a one-step ligation. **(C)** Structure of destination vectors used in the CRISPR/Cas9 system.

Three sgRNAs guiding sequences were inserted into the SK-gRNA vector (Wang et al., 2015) between the two *Aar* I sites using annealed oligonucleotides (we named the resulting three vectors SK-gRNA1, SK-gRNA2, and SK-gRNA3). The pC1300-Cas9 vector contains the codon-optimized *Streptococcus pyogenes* Cas9 gene and the *Kpn* I and *BamH* I isocaudomer enzyme sites, which are designed to allow integration of gRNA cassettes. The SK-gRNA vector also contains isocaudomer restriction enzyme sites, namely *Spel/ Xba* I, *Xho* I*/Sal* I, and *BamH* I/BgI II sites (Figure 2A). The steps of vector construction are shown in Figure 2B. The three vectors (SK-gRNA1, SK-gRNA2, and SK-gRNA3) were digested separately with restriction enzymes, and then inserted into the pC1300-2 × 35S-Cas9 and pC1300-MAS-Cas9 binary vector between the *Kpn* I and *Bam*H I sites by one-step ligation (Wang et al., 2015). The constructed vectors were named *PagPDS-*2 × 35S-Cas9, and *PagPDS*-MAS-Cas9, respectively. The final vector diagram is shown in Supplementary Figure 1 and the Cas9 sequence in Supplementary Sequence 2. The construct was sequenced and the expected sequences confirmed.

### Transformation and Regeneration

The vector containing CRISPR/Cas9 with gRNA expression cassettes was then transformed into the *Agrobacterium* tumefaciens strain GV3101 (Han et al., 2000; Li et al., 2017; An et al., 2020b). Briefly, *Agrobacterium* cells harboring the vectors were harvested by centrifugation and then resuspended to OD_600_ = 0.3–0.4. Poplar 84K leaf disks were soaked for 20 min on a shaker with the resuspended cells at room temperature. The inoculated leaf disks were co-cultivated at 22°C in the dark for 2 to 3 days. The leaf discs were then washed with sterile double-distilled water and cultured on a callus-induction medium for 10 to 30 days in the dark. The transgenic lines were selected by culturing on medium supplemented with hygromycin (2.0 mg/L). The gene-edited plants were vegetatively propagated in half-strength Murashige and Skoog medium (pH 5.7) containing 0.8% (w/v) agar at room temperature under the light intensity of 50 μmol m^−2^ s^−1^ and a 16/8 h (light/dark) photoperiod.

### Detection of Mutations

For analysis of the mutations of edited *PagPDS* in transgenic T_0_ plants, genomic DNA was extracted from stable transgenic or wild-type plants using CTAB as described above. The genomic DNAs were used as templates to amplify the endogenous *PagPDS* fragment by Polymerase Chain Reaction (PCR). The primers used for PCR were PagPDS_Mut_F: AACTGGGTATGCGAAGACTTCC and PagPDS_Mut_R: GATTTCATCACCTGCACCAGCAAT. The primers amplified the region spanning the three target sites of the Cas9 system (target 1: 5′-GAGTGCATTGAACTTGAGCTGG-3′; target 2: 5′-CCAAGACCGGACCTTGATAACA-3′; target 3: 5′-CCGGACCTTGATAACACGGTGA-3′). The PCR products were amplified and cloned into the pMD18-T Simple vector as mentioned above for Sanger sequencing to evaluate the editing outcomes of CRISPR/Cas9 transfection. Finally, individual sequences were aligned to the wild-type sequence using SnapGene to determine the mutations effects on predicted modified peptide sequences.

## Results

### CRISPR/Cas9 Targeted Mutagenesis in *PagPDS*

To establish a new CRISPR/Cas9 genome editing system in poplars, we cloned the phytoene desaturase gene 8 from the hybrid poplar 84K and selected three target sites with 5′-NGG-3′ PAMs of two exons. We named the three target sites T1, T2, and T3 (Figure 1A). The multiple CRISPR RNA (crRNA) cassettes were driven by the *Arabidopsis* RNA polymerase III promoter AtU6-26 while Cas9 was driven by the 2 × 35S promoter or MAS promoter in pCambia1300 binary vectors (Figure 2A). The target sequence was confirmed by amplification and sequencing. The three gRNAs were inserted into the *pC1300-2* × *35S-Cas9* and *pC1300-MAS-Cas9* binary vectors between the *Kpn* I and *BamH* I sites by a one-step ligation (Figure 2B). Two constructs (Figure 2C) were introduced into the poplar 84K via *Agrobacterium*-mediated transformation. The transgenic plants were selected by culture on $\frac{1}{2}$ MS medium supplemented with hygromycin and grown with a light and dark cycles of 16/8 h.

### Phenotypes of *PagPDS-2 × 35S-Cas9* Transgenic Plants

**Figure 3:**
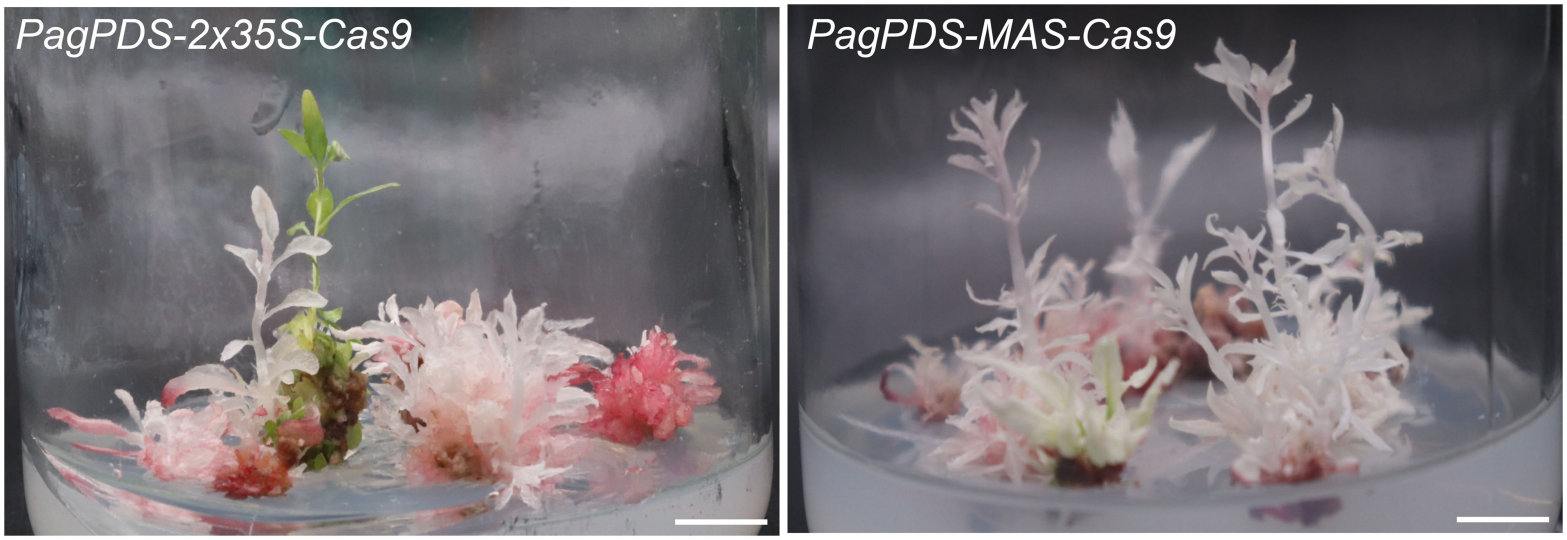
Phenotypes of the *PagPDS-2* × *35S/MAS*-Cas9-mediated transgenic plants. Scale bar = 1 cm.

**Figure 4:**
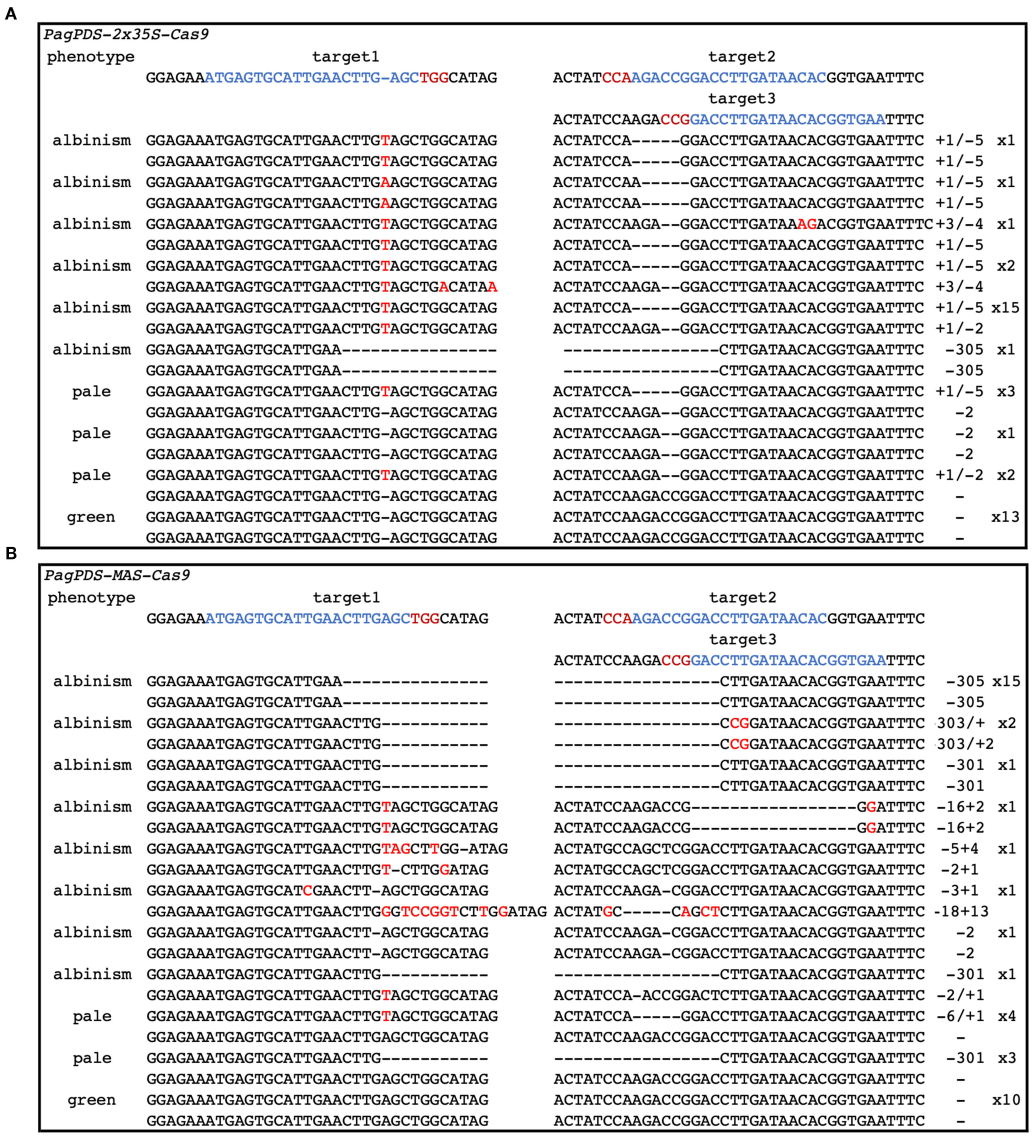
CRISPR/Cas9-mediated gene editing in transgenic poplar plants. **(A)** Editing situation in *PagPDS-2* × *35S-Cas9* plants. **(B)** Editing situation in *PagPDS-MAS-Cas9* plants.

Forty positive candidate transgenic lines were obtained for *PagPDS-2* × *35S-Cas9* and confirmed by DNA PCR amplification. Both biallelic homozygous and heterozygous mutants of *PDS* are known to exhibit albino phenotypes (Fan et al., 2015). Here, albino seedlings were observed, confirming that mutations were generated at the *PDS* gene (Figure 3). To evaluate the simultaneous editing of multiple target sites, the genomic DNA of 40 transgenic plants was extracted independently, and the targeted sequences of *PDS* were amplified by PCR. The sequencing results showed 67.5% (27 out of 40) mutation rate. Out of 40 albino seedlings, 77.8% (21 out of 27) were albinos and 22.2% (6 out of 27) showed pale green phenotype (Table 1). At the T1 site, all seedlings carried mutations, and 53.75% of the mutations were a mononucleotide insertion. At T2/T3 loci, deletions were noted ranging from 2 to 5 nt near the PAM. Gene editing data are presented in Figure 4A. At the T1 locus, five plants had a mutation in one allele while 21 plants had mutations in both alleles, including 20 homozygous mutations and 1 biallelic mutations. At T2/T3 loci, the mutation rate was 67.5% (27 out of 40), including 21 biallelic mutations and four homozygous mutations (Table 2).

**Table 1:** Determination of mutation rate in transgenic T_0_ poplar plants generated with the *PagPDS-2* × *35S/MAS*-Cas9 system.

**Target gene**	**Number of plants examined**	**Number of plants with mutations**	**Mutation rate (%)**	**Albinism phenotype**		**Pale green phenotype**	
				**Number**	**%**	**Number**	**%**
*PagPDS-2 × 35S-Cas9*	40	27	67.5	21	77.8	6	22.2
*PagPDS-MAS-Cas9*	40	30	75	23	76.7	7	23.3
CK	40	0	0	ND	ND	ND	ND

*CK, Empty vector; ND, not determined*.

**Table 2:** Summary of the mutation types at each target site.

	***PagPDS-2×35S-Cas9***		***PagPDS-MAS-Cas9***	
	**T1**	**T2/T3**	**T1**	**T2/T3**
Biallelic mutation	1	21	3	2
Homozygous	20	4	19	20
Heterozygous	5	2	7	7
Unmodified	14	13	11	11
Mutation rate	65%	67.5%	72.5%	72.5%

### Phenotypes of *PagPDS-MAS-Cas9* Transgenic Plants

To explore the activity of the MAS promoter in poplars, we used the same method to construct a vector with MAS driving expression of Cas9. This construct was introduced into poplar 84K resulting in 40 transgenic lines, of which30 were albino. In the MAS-Cas9 system, the mutation rate was 75% (30 out of 40), which was higher than that of the 2 × 35S-Cas9 system. 76.7% (23 of 30) of mutant plants were albinos and 23.3% (7 out of 30) showed a pale green phenotype (Table 1). Sequencing of the albino plants was used to define the resulting mutations. Large deletions were found between T1 and T2/T3 (Figure 4B). At mutation site T1, 29 plants (72.5%) had at least one allele edited while 22 plants had the edited mutations in both alleles, including 19 homozygous mutations and 3 biallelic mutations. At the T2/T3 loci, the mutation rate was 72.5%, including 20 homozygous mutations and 2 biallelic mutations (Table 2).

## Discussion

In this study, we established a simplified method for creating mutations with high frequency directly in the model poplar 84K genotype. We used two vectors to construct an engineered binary vector based on the classical isocaudomer technique to simplify the procedures. With appropriate restriction enzyme sites, as many as three gRNAs or more can be assembled together in one step in a single ligation reaction, which enables creating mutations in multiple target sites within a single plant. This system both simplifies the procedures for gene editing, and also increases the frequency of mutations.

Our ability to successfully target multiple sites of the *PagPDS* genes in *Populus* make demonstrates the ability to edit multiple genes at the same time, including duplicated homologous genes. Gene duplication is common in poplar and other trees, so the simple system could be applied to edit all homologs in trees. In order to better compare the editing efficiency with the binary pYLCRIPSR/Cas9, three target sites were selected using the criteria of Fan et al. (2015). The mutation rate in our system is improved to higher rate, 67.5% in CaMV 35S promoter-based system and 75% in pC1300-MAS-Cas9 system than the reported mutation rate (51.7%) in *Populus tomentosa Carr* (Fan et al., 2015). Indeed in our pC1300-MAS-Cas9 system, there is high editing efficiency, which is of practical importance as it reduces the work and effort of producing and evaluating enough transgenic lines to recover desired mutations. Based on previous studies, the MAS promoter increases the expression of gene fusions in tobacco and maize (Ni et al., 1995; Lee et al., 2007). It is thus likely that the MAS promoter promoted the expression of Cas9 in our experiments in poplar, enriching the variety and efficiency of edits.

Our a high-efficiency genome-editing tool of pC1300-MAS-Cas9 will facilitate gene functional research in woody tree species. In future studies we will directly test whether the PC1300-MAS-Cas9 has high editing efficiency in other woody plants and trees.

## Data Availability Statement

The original contributions presented in the study are included in the article/Supplementary Material, further inquiries can be directed to the corresponding author/s.

## Author Contributions

YA, JD, and CW designed the research and wrote the paper. YA, CW, JY, and YG performed the research. YA and JY analyzed data. All authors reviewed the manuscript.

## Conflict of Interest

The authors declare that the research was conducted in the absence of any commercial or financial relationships that could be construed as a potential conflict of interest.
