# Supplementary material for: An Improved CRISPR/Cas9 System for Genome Editing in Populus by Using Mannopine Synthase (MAS) Promoter
Source: Front Plant Sci. 2021 Jul 12;12:703546. doi: 10.3389/fpls.2021.703546 (PMC8311491; doi:10.3389/fpls.2021.703546)
Supplement: Supplementary file 1 [file Data_Sheet_1.DOCX]

**Supplementary**

**Supplementary sequence 1: Sequence of the MAS promoter**

TTTTCAAATCAGTGCGCAAGACGTGACGTAAGTATCCGAGTCAGTTTTTATTTTTCTACTAATTTGGTCGTTTATTTCGGCGTGTAGGACATGGCAACCGGGCCTGAATTTCGCGGGTATTCTGTTTCTATTCCAACTTTTTCTTGATCCGCAGCCATTAACGACTTTTGAATAGATACGCTGACACGCCAAGCCTCGCTAGTCAAAAGTGTACCAAACAACGCTTTACAGCAAGAACGGAATGCGCGTGACGCTCGCGGTGACGCCATTTCGCCTTTTCAGAAATGGATAAATAGCCTTGCTTCCTATTATATCTTCCCAAATTACCAATACATTACACTAGCATCTGAATTTCATAACCAATCTCGATACACCAAATCG

**Supplementary sequence 2: Sequence of Cas9**

ATGGCCCCTAAGAAGAAGAGAAAGGTCGGTATTCACGGCGTTCCTGCGGCGATGGACAAGAAGTATAGTATTGGTCTGGACATTGGGACGAATTCCGTTGGCTGGGCCGTGATCACCGATGAGTACAAGGTCCCTTCCAAGAAGTTTAAGGTTCTGGGGAACACCGATCGGCACAGCATCAAGAAGAATCTCATTGGAGCCCTCCTGTTCGACTCAGGCGAGACCGCCGAAGCAACAAGGCTCAAGAGAACCGCAAGGAGACGGTATACAAGAAGGAAGAATAGGATCTGCTACCTGCAGGAGATTTTCAGCAACGAAATGGCGAAGGTGGACGATTCGTTCTTTCATAGATTGGAGGAGAGTTTCCTCGTCGAGGAAGATAAGAAGCACGAGAGGCATCCTATCTTTGGCAACATTGTCGACGAGGTTGCCTATCACGAAAAGTACCCCACAATCTATCATCTGCGGAAGAAGCTTGTGGACTCGACTGATAAGGCGGACCTTAGATTGATCTACCTCGCTCTGGCACACATGATTAAGTTCAGGGGCCATTTTCTGATCGAGGGGGATCTTAACCCGGACAATAGCGATGTGGACAAGTTGTTCATCCAGCTCGTCCAAACCTACAATCAGCTCTTTGAGGAAAACCCAATTAATGCTTCAGGCGTCGACGCCAAGGCGATCCTGTCTGCACGCCTTTCAAAGTCTCGCCGGCTTGAGAACTTGATCGCTCAACTCCCGGGCGAAAAGAAGAACGGCTTGTTCGGGAATCTCATTGCACTTTCGTTGGGGCTCACACCAAACTTCAAGAGTAATTTTGATCTCGCTGAGGACGCAAAGCTGCAGCTTTCCAAGGACACTTATGACGATGACCTGGATAACCTTTTGGCCCAAATCGGCGATCAGTACGCGGACTTGTTCCTCGCCGCGAAGAATTTGTCGGACGCGATCCTCCTGAGTGATATTCTCCGCGTGAACACCGAGATTACAAAGGCCCCGCTCTCGGCGAGTATGATCAAGCGCTATGACGAGCACCATCAGGATCTGACCCTTTTGAAGGCTTTGGTCCGGCAGCAACTCCCAGAGAAGTACAAGGAAATCTTCTTTGATCAATCCAAGAACGGCTACGCTGGTTATATTGACGGCGGGGCATCGCAGGAGGAATTCTACAAGTTTATCAAGCCAATTCTGGAGAAGATGGATGGCACAGAGGAACTCCTGGTGAAGCTCAATAGGGAGGACCTTTTGCGGAAGCAAAGAACTTTCGATAACGGCAGCATCCCTCACCAGATTCATCTCGGGGAGCTGCACGCCATCCTGAGAAGGCAGGAAGACTTCTACCCCTTTCTTAAGGATAACCGGGAGAAGATCGAAAAGATTCTGACGTTCAGAATTCCGTACTATGTCGGACCACTCGCCCGGGGTAATTCCAGATTTGCGTGGATGACCAGAAAGAGCGAGGAAACCATCACACCTTGGAACTTCGAGGAAGTGGTCGATAAGGGCGCTTCCGCACAGAGCTTCATTGAGCGCATGACAAATTTTGACAAGAACCTGCCTAATGAGAAGGTCCTTCCCAAGCATTCCCTCCTGTACGAGTATTTCACTGTTTATAACGAACTCACGAAGGTGAAGTATGTGACCGAGGGAATGCGCAAGCCCGCCTTCCTGAGCGGCGAGCAAAAGAAGGCGATCGTGGACCTTTTGTTTAAGACCAATCGGAAGGTCACAGTTAAGCAGCTCAAGGAGGACTACTTCAAGAAGATTGAATGCTTCGATTCCGTTGAGATCAGCGGCGTGGAAGACAGGTTTAACGCGTCACTGGGGACTTACCACGATCTCCTGAAGATCATTAAGGATAAGGACTTCTTGGACAACGAGGAAAATGAGGATATCCTCGAAGACATTGTCCTGACTCTTACGTTGTTTGAGGATAGGGAAATGATCGAGGAACGCTTGAAGACGTATGCCCATCTCTTCGATGACAAGGTTATGAAGCAGCTCAAGAGAAGAAGATACACCGGATGGGGAAGGCTGTCCCGCAAGCTTATCAATGGCATTAGAGACAAGCAATCAGGGAAGACAATCCTTGACTTTTTGAAGTCTGATGGCTTCGCGAACAGGAATTTTATGCAGCTGATTCACGATGACTCACTTACTTTCAAGGAGGATATCCAGAAGGCTCAAGTGTCGGGACAAGGTGACAGTCTGCACGAGCATATCGCCAACCTTGCGGGATCTCCTGCAATCAAGAAGGGTATTCTGCAGACAGTCAAGGTTGTGGATGAGCTTGTGAAGGTCATGGGACGGCATAAGCCCGAGAACATCGTTATTGAGATGGCCAGAGAAAATCAGACCACACAAAAGGGTCAGAAGAACTCGAGGGAGCGCATGAAGCGCATCGAGGAAGGCATTAAGGAGCTGGGGAGTCAGATCCTTAAGGAGCACCCGGTGGAAAACACGCAGTTGCAAAATGAGAAGCTCTATCTGTACTATCTGCAAAATGGCAGGGATATGTATGTGGACCAGGAGTTGGATATTAACCGCCTCTCGGATTACGACGTCGATCATATCGTTCCTCAGTCCTTCCTTAAGGATGACAGCATTGACAATAAGGTTCTCACCAGGTCCGACAAGAACCGCGGGAAGTCCGATAATGTGCCCAGCGAGGAAGTCGTTAAGAAGATGAAGAACTACTGGAGGCAACTTTTGAATGCCAAGTTGATCACACAGAGGAAGTTTGATAACCTCACTAAGGCCGAGCGCGGAGGTCTCAGCGAACTGGACAAGGCGGGCTTCATTAAGCGGCAACTGGTTGAGACTAGACAGATCACGAAGCACGTGGCGCAGATTCTCGATTCACGCATGAACACGAAGTACGATGAGAATGACAAGCTGATCCGGGAAGTGAAGGTCATCACCTTGAAGTCAAAGCTCGTTTCTGACTTCAGGAAGGATTTCCAATTTTATAAGGTGCGCGAGATCAACAATTATCACCATGCTCATGACGCATACCTCAACGCTGTGGTCGGAACAGCATTGATTAAGAAGTACCCGAAGCTCGAGTCCGAATTCGTGTACGGTGACTATAAGGTTTACGATGTGCGCAAGATGATCGCCAAGTCAGAGCAGGAAATTGGCAAGGCCACTGCGAAGTATTTCTTTTACTCTAACATTATGAATTTCTTTAAGACTGAGATCACGCTGGCTAATGGCGAAATCCGGAAGAGACCACTTATTGAGACCAACGGCGAGACAGGGGAAATCGTGTGGGACAAGGGGAGGGATTTCGCCACAGTCCGCAAGGTTCTCTCTATGCCTCAAGTGAATATTGTCAAGAAGACTGAAGTCCAGACGGGCGGGTTCTCAAAGGAATCTATTCTGCCCAAGCGGAACTCGGATAAGCTTATCGCCAGAAAGAAGGACTGGGACCCGAAGAAGTATGGAGGTTTCGACTCACCAACGGTGGCTTACTCTGTCCTGGTTGTGGCAAAGGTGGAGAAGGGAAAGTCAAAGAAGCTCAAGTCTGTCAAGGAGCTCCTGGGTATCACCATTATGGAGAGGTCCAGCTTCGAAAAGAATCCGATCGATTTTCTCGAGGCGAAGGGATATAAGGAAGTGAAGAAGGACCTGATCATTAAGCTTCCAAAGTACAGTCTTTTCGAGTTGGAAAACGGCAGGAAGCGCATGTTGGCTTCCGCAGGAGAGCTCCAGAAGGGTAACGAGCTTGCTTTGCCGTCCAAGTATGTGAACTTCCTCTATCTGGCATCCCACTACGAGAAGCTCAAGGGCAGCCCAGAGGATAACGAACAGAAGCAACTGTTTGTGGAGCAACACAAGCATTATCTTGACGAGATCATTGAACAGATTTCGGAGTTCAGTAAGCGCGTCATCCTCGCCGACGCGAATTTGGATAAGGTTCTCTCAGCCTACAACAAGCACCGGGACAAGCCTATCAGAGAGCAGGCGGAAAATATCATTCATCTCTTCACCCTGACAAACCTTGGGGCTCCCGCTGCATTCAAGTATTTTGACACTACGATTGATCGGAAGAGATACACTTCTACGAAGGAGGTGCTGGATGCAACCCTTATCCACCAATCGATTACTGGCCTCTACGAGACGCGGATCGACTTGAGTCAGCTCGGGGGGGATAAGAGACCAGCGGCAACCAAGAAGGCAGGACAAGCGAAGAAGAAGAAG

**Supplementary Figure 1：**Final vector diagram (a) pC1300-2x35S-Cas9-PDSg1-g2-g3

(b) pC1300-MAS-Cas9-PDSg1-g2-g3

**
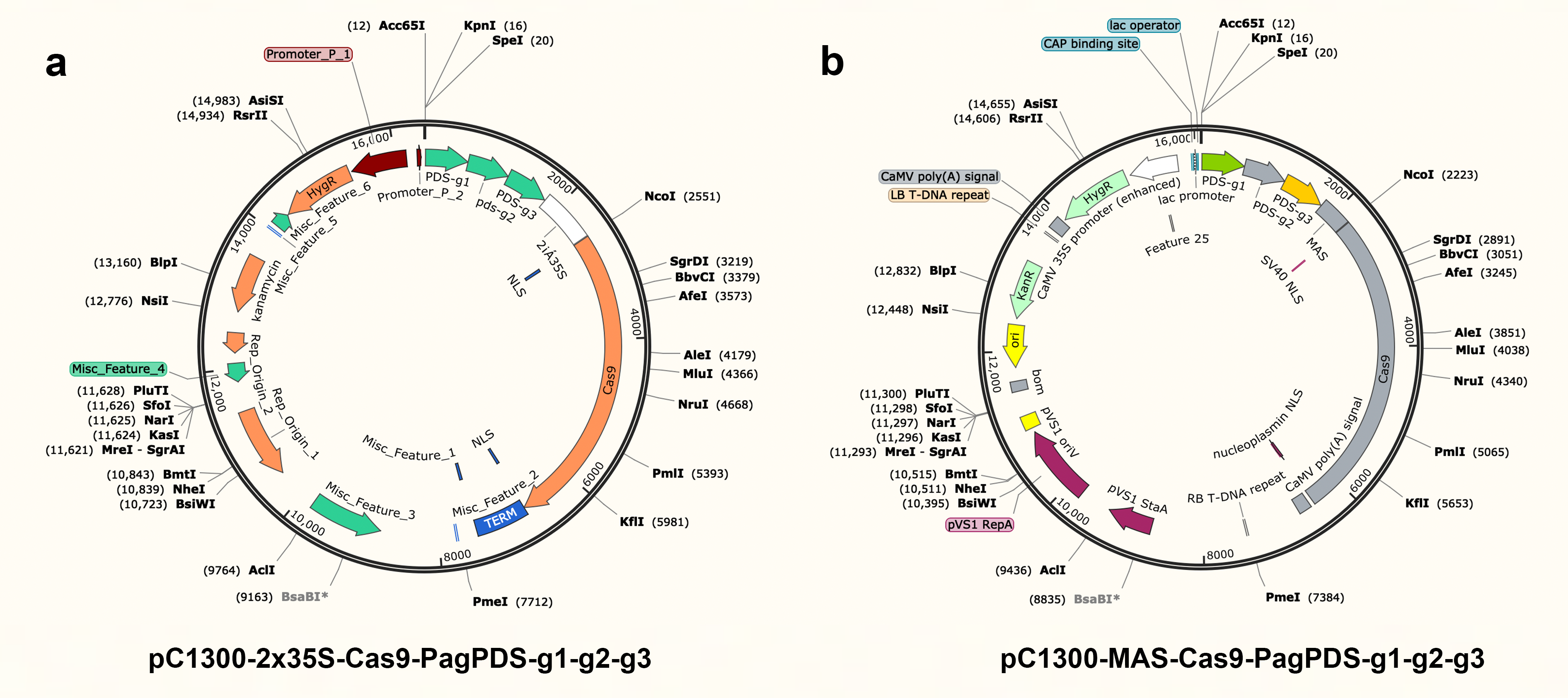
**
